# Detection of Fractal Behavior in Temporal Series of Synaptic Quantal Release Events: A Feasibility Study

**DOI:** 10.1155/2012/704673

**Published:** 2012-08-14

**Authors:** Jacopo Lamanna, Antonio Malgaroli, Sergio Cerutti, Maria G. Signorini

**Affiliations:** ^1^Department of Bioengineering, Politecnico di Milano, Piazza Leonardo da Vinci 32, 20133 Milano, Italy; ^2^Neurobiology of Learning Unit, Scientific Institute San Raffaele and Università Vita e Salute San Raffaele, Via Olgettina 58, 20132 Milano, Italy

## Abstract

Since the pioneering work of Fatt and Katz at the neuromuscular junction (NMJ), spontaneous synaptic release (minis), that is, the quantal discharge of neurotransmitter molecules which occurs in the absence of action potentials, has been unanimously considered a memoryless random Poisson process where each quantum is discharged with a very low release probability independently from other quanta. When this model was thoroughly tested, for both population and single-synapse recordings, some clear evidence in favor of a more complex scenario emerged. This included short- and long-range correlation in mini occurrences and divergence from mono-exponential inter-mini-interval distributions, both unexpected for a homogeneous Poisson process, that is, with a rate parameter that does not change over time. Since we are interested in accurately quantifying the fractal exponent *α* of the spontaneous neurotransmitter release process at central synaptic sites, this work was aimed at evaluating the sensitivity of the most established methods available, such as the periodogram, the Allan, factor and the detrended fluctuation analysis. For this analysis we matched spontaneous release series recorded at individual hippocampal synapses (single-synapse recordings) to generate large collections of simulated quantal events by means of a custom algorithm combining Monte Carlo sampling methods with spectral methods for the generation of 1/*f* series. These tests were performed by varying separately: (i) the fractal exponent *α* of the rate driving the release process; (ii) the distribution of intervals between successive releases, mimicking those encountered in single-synapse experimental series; (iii) the number of samples. The aims were to provide a methodological framework for approaching the fractal analysis of single-unit spontaneous release series recorded at central synapses.

## 1. Introduction

Based on classical work at the neuromuscular junction (NMJ) and at other peripheral terminals [1], individual synaptic release sites have been always assumed to behave independently and to discharge quanta at a very low and stable rate. Regrettably, based on the large number of contrasting evidence which has accumulated since then, this memory-less description of the spontaneous release process might not always apply to CNS synapses [2–4]. For example, it has been shown that the occurrence of minis does exhibit long-term correlations [2], a synaptic memory that might be expressed, for example, by a correlating phenomenon such as a local changes in intracellular calcium at the active zone, where release sites are located.

The most popular mean to study the statistics of spontaneous release is to analyze the distributions of inter-quanta intervals from large data sets [1]. These are almost invariably obtained by recording from a population of synapses or active zones (population recordings). Although for a homogeneous *Poisson* process, characterized by a single and stable *Poisson *rate, the predicted frequency distribution of intervals should display a mono-exponential profile, some clear divergences have been reported [2–4].

Could the reported divergence from the mono-exponential case arise because of temporal averaging of the activity of many different synapses, each one discharging quanta with its characteristic Poisson rate? Clearly, if we consider that each synapse generates spontaneous events around its specific Poisson rate, if this rate is constant and the synaptic units all independent, then the compound process for the population activity will also be Poisson [3].

Can a set of different synapses be considered as an independent source of quanta? In some experimental conditions, this is likely to happen, for example, when the generation of action potentials is prevented by tetrodotoxin and/or when a spontaneous or evoked calcium raise is prevented by calcium buffers or by specific compounds. 

The divergence from mono-exponential interval distributions [2–4] and the finding of short- and long-range correlations in mini occurrences [2, 5] might then arise from a nonindependent behavior of quantal discharges because of some forms of molecular or physical correlations between quanta at the active zone or because of some unknown mechanism of correlation between neighboring presynaptic terminals when impinging upon the same postsynaptic neuron.

To address this critical issue, the most important requirement would then be to study the dynamics of spontaneous quantal discharges at individual central synapses. Indeed, single-synapse recordings from hippocampal synapses were previously used to analyze the statistics of the discharge process [4–6]. Quantal discharges gathered by single-bouton recordings, despite the small sample size, were always characterized by multiexponential distributions of mini-intervals [4]. This behavior could not be accounted by random coincidences and was found to be related to the occurrence of short epochs of multiple quanta releases. This release modality could also be clearly detected in population whole-cell recordings [3, 4]. Using the latter type of electrophysiological recordings, sampling the activity of the very many synapses, it was also found that the quantal release rate spectrum displayed a 1/*f* power law [2, 5]. A scaling exponent close to 1, in the rate spectrum of quantal releases detected by population recordings, indicates that the long-memory process or 1/*f* behavior presumably reflects either a time-dependent activity correlation among different terminals or a general synaptic behavior where at each individual synaptic active zone quanta do indeed correlate.

Lowen and colleagues modeled quantal release by a fractal-lognormal noise-driven Poisson point process (FLNP), that is, a stochastic-rate Poisson process (DSPP) driven by a fractal-lognormal noise [2]. Interestingly, on the basis of biophysical considerations, they suggested that the rate process triggering quantal releases can be modulated by 1/*f* oscillations of membrane voltage through a logarithmic transform [2]. Subthreshold membrane voltage oscillations might spread far away along dendrites and axonal networks, hence they might represent an effective mechanism of activity correlation for neighboring terminals. In this respect, it is worth considering that population release activity not only cannot be used to distinguish between one-synapse and multisynapse correlation mechanisms, but also, because of the temporal superimposition of a large number of release series, might obscure the real temporal characteristics of these correlations.

To better address this specific issue, we therefore have begun the analysis of the frequency characteristics of quantal releases seen with single-bouton recordings. In these experiments, characterized by interevent intervals distributions which were always best-fitted by sums of exponential functions, the frequency analysis revealed a clear 1/*f* power law in the rate spectrum which was resistant to intervals shuffling [5]. In the present paper, as further validation, we have tested for the application of a few standard methods for fractal analysis, that is the periodogram, the Allan factor, and the DFA method.

The goal was to characterize the sensitivity of these methods when applied to spontaneous release series gathered from single synapses, usually characterized by a small sample size and a non-Poisson interval distribution. We generated simulated series of release events by combining Monte Carlo sampling methods with an integrate-and-fire model. The idea was to mimic the rate and the behavior of interevent intervals seen in single synapse recordings. Based on this input, we have determined the ability of the above methods in searching and accurately quantifying the 1/*f* behavior.

## 2. Methods

### 2.1. Interevent Interval Distributions and Histograms Generation

As previously reported [4], single-synapse recordings strongly indicate that the distribution of intervals between successive releases of quanta diverges from the exponential form. The sum of two or even three exponential functions is actually needed in order to fit interevent interval histograms. This kind of interval distribution can be referred to as hyperexponential. For sake of simplicity we limited our computational survey to the “biexponential” case: the probability density function of the interevent interval was taken as

(1) $$\begin{array}{c} {\text{pdf}\left(T\geq t\right)=\;a}_{f}\lambda_{f}e^{-\lambda_{f}t}+a_{s}\lambda_{s}e^{-\lambda_{s}t}, \end{array}$$

where *a*_*f*_, *a*_*s*_ are the fast and slow relative areas, respectively, and *λ*_*f*_, *λ*_*s*_ are the fast and slow rate constants of the two decaying exponentials, respectively.

This form of pdf was used for the simulation of intervals by means of Monte Carlo (MC) sampling. Although we know the analytical form of the pdf, we choose to generate interevent interval histograms of the simulated data according to the log binned representation [7, 8]. This let us maintain a straightforward comparison of our simulated data with both the real and simulated data of single-synapse recordings previously reported by our group [3, 4]. For such a representation, intervals between successive simulated quanta (taken as peak-to-peak distances) were binned according to the logarithm of their durations. This produces an increase in bin width (bw_*i*_) as interval duration increases. The number of intervals *n*_*i*_ falling in each bin (bin content) was divided by bin width in order to obtain a normalized bin content *N*_*i*_ = *n*_*i*_/bw_*i*_.

Histograms plot *N*_*i*_ as a function of bin center *c*_*i*_ (the center of the *i*th bin) on doubly-logarithmic scale. This representation has the twice advantage of improving visual perception and providing better fitting of hyperexponential distributions when distribution tails contain very few events, as it will be shown in Section 4 (see [7, 8]).

### 2.2. Simulated Series of Quantal Releases following a Power-Law Rate and a Biexponential Distribution of Intervals

Our computational aim was to generate a wide set of simulated release series matching two experimentally evidenced features which are of great interest for us.

- A power-law spectrum of the release rate.
- A distribution of intervals assuming the hyperexponential form.

Furthermore, since the formulation of a new and complete model based on physiological knowledge falls outside the scope of this work, these simulations should maintain assumptions about the process generation model as limited as possible. As previously discussed, several lines of evidence exist for refusing the standard homogeneous Poisson model for describing the release process in the case of both single-synapse and population-of-synapses recordings. A general Doubly Stochastic Poisson Point Process (DSPP) could be assumed, with an instantaneous stochastic rate characterized by power-law spectrum, as experimentally observed [2, 5]. Since a biexponential distribution of the interevent interval was taken here as a satisfying model for fitting the histograms generated with experimental data, the main question is if such a DSPP model would be able to generate this interval distribution. We faced this problem by adopting a black-box approach, thus overcoming computational bottlenecks. Indeed, the coefficient of variation (CV) of the DSPP driving rate is generally kept low in order to maintain it nonnegative for most of the simulation length. Furthermore, this rate should be stationary for analytical purposes.

For all these reasons, we opted for developing a custom simulation algorithm which is able to convert, my means of a nonlinear transform, a 1/*f* ^*α*^ rate signal into a point process whose interval distribution matches the biexponential form. 

The rate process *λ*(*t*) is the first source of randomness for the simulated process and can be obtained by generating a fractal Gaussian process (fGp) with power-law spectrum of the form 1/*f* ^*α*^. To get good approximations of the fGp, a modified version of the spectral synthesis method (SSM) proposed by Saupe was implemented [9]. The SSM is the purest interpretation of the concept of fractal Brownian motion (fBm) and fractal Gaussian noise (fGn). SSM perfectly outlines their spectral features by imposing a power-law random-phases spectrum with the desired value of the scaling exponent *α*. To the generated power spectrum, the inverse fast Fourier transform was applied to get the fGp time series and unwanted periodicities were avoided by discarding 15/16 of the samples.

For obtaining a discrete sequence of releases *t*(*n*), we implemented an “integrate-and-fire” approach: the rate process *λ*(*t*) was integrated over time until the integrated series *φ*(*t*) reached the unit (occurrence of a release event), then *φ*(*t*) was reset to zero. Some modifications of *λ*(*t*) were performed for assuring the method worked properly. The steps were as follows.

- *λ*(*t*) was normalized so that its standard deviation was equal to 0.6.
- *λ*(*t*) was then transformed into a fractal-lognormal noise by means of an exponential transform. As previously reported in [10], this transformation maintains the spectral features of the signal only for small values of the variance. This is the reason why we fixed *std* equal to 0.6, in accordance with literature and after experimental validation.
- The amplitude of *λ*(*t*) was normalized again for obtaining an integral value equal to the expected number of events *N*.

After this first simulation stage, the temporal locations of the events precisely reflect the fractal features of the underlying rate. Nevertheless, the intervals between the events are not expected to assume the whished biexponential distribution. This second source of randomness was then achieved by replacing the “fractal” set of intervals generated by the “integrate-and-fire” approach with a different set of intervals which are separately generated by Monte Carlo (MC) sampling of the biexponential probability density functions. These pdfs were chosen according to different sets of parameters.

This second stage implemented by our algorithm requires the replacement of the “fractal” intervals with a set of intervals generated by MC sampling. Since MC sampling introduces some error in sampling the ideal pdf due to limited sample size, we avoided any further increase of this error. Nevertheless, for achieving a fractal rate, the sequential order of the MC intervals was chosen to follow the relative order of the “fractal” intervals. As a perfect match is impossible since the intervals of the two sets are very different in their distribution, the algorithm allows for a “tolerance window” of about 10 s. In other words, we choose as a good match the first interval of the MC set, randomly extracted, which was 5 s longer or shorter than the original “fractal” interval. Although this “tolerance window” may appear as very large, it let us maintain the fractal features of the rate for time scales longer than 10 s. This is a valuable tradeoff for our analysis purposes. 

The resulting simulated series is a point process whose generation model is not known actually, still it is able to reflect the two features of interest: (i) a power-law spectrum of the releases rate for time-scales higher than 10 s *c.ca*; (ii) an interevent interval distribution given by the biexponential model whose parameters are known *a priori*. As for the latter, only one parameter of the biexponential probability density function was varied: the area of the fast exponential component *a*_*f*_. According to the available experimental data, the following parameters were set: *λ*_*f*_ = 10 ms, *λ*_*s*_ = 1 s, *a*_*f*_ = 2, 15, 50, 85%, *a*_*s*_ = 1 − *a*_*f*_ and *α* = 0, 0.5, 1, 1.5, 2. For each pair of independent parameters (*a*_*f*_, *α*), 20 sequences of *N* = 10^4^ simulated releases were generated. These values of *α* were selected for their physical importance, since they can be related to uncorrelated white noise, flicker noise, and Brownian behavior.

## 3. Methods for the Quantification of Fractal Behavior

### 3.1. Periodogram

A method of choice for assessing fractal behavior is the estimation of the power spectral density (PSD) of the point process. PSD is obtained by computing the periodogram (PG) of the point process by means of the so-called count-based PG. As previously reported [9], the count-based PG outclasses interval-based PG for estimation errors (the error bias in particular) and is the only spectral choice that preserves the physical significance of the frequency axis.

For obtaining this PG, we implemented the algorithm proposed in [10]. The length of the series (the time of the last release, *t*_max⁡_) is divided in contiguous windows of length *T*. A count series {*W*_*i*_} is then obtained by further dividing each window in *M* segments of 0.1 s (taken as fixed resolution) and counting the number of events falling in each segment.

The PG is then obtained for each window as $S_{w}(f)=1/M{\left|\tilde{W}(f)\right|}^{2}$, where $\tilde{W}(f)$ is the discrete Fourier transform of the count series {*W*_*i*_}. Then, an single PG, *S*(*f*), is obtained my averaging all the windows-related PGs, *S*_*w*_(*f*). This PG is an accurate estimate of the PSD of the point process in the range: 1/*T* ÷ *M*/2*T* Hz.

The count-based PG follows a power-law of the form 1/*f* ^*α*^ in the low and medium frequency range for fractal-rate point processes. PG introduces a bias at higher frequencies, since the fine time resolution information is lost due to the finite counting window size (0.1 s in our case). However, this bias is negligible as the *α* estimation is usually performed on the low frequencies.

To estimate the fractal exponent using the PSD on simulated point process (20 realizations, parameters *a*_*f*_ and *α*), we proceeded as follows: (i) we computed the PG on each realization with a common *t*_max⁡_ equal to the maximum release time among all the realizations (the variance of this value among the realizations is small, since each set of intervals is generated with same pdf and *N* = 10^4^); (ii) we divided the PGs by the DC value (*f* = 0) for comparability; (iii) we computed the logarithm (basis 10) of the single PGs and then averaged them to obtain an average logarithmic PG (we will call it $\overset{-}{\text{logPG}}$) relative to the whole set of realizations; (iv) excluding the DC value for convention and imposing a cutoff frequency manually, we obtained an accurate estimate for the fractal exponent $\hat{\alpha }$ relative to a simulated process by linear least mean square regression of the $\overset{-}{\text{logPG}}$ versus log⁡_10_(*f*) curve, limiting the fitting to the frequency range manually selected.

### 3.2. Allan Factor (AF)

The Allan factor (AF) is a normalized form of Allan variance. For a point processes, it is computed as *A*(*T*) = *E*[(*Z*_*k*+1_−*Z*_*k*_)^2^]/2*E*[*Z*_*k*_], where *Z*_*k*_ is the count series obtained with counting window length *T* [11]. The AF for a fractal point process assumes the power-law form *A*(*T*) = 1 + (*T*/*T*_0_)^*α*^, where *T*_0_ is the fractal onset time. On a doubly-logarithmic scale, this function can be fitted by linear regression from *T*_0_ on. As in the PG case, we decided to perform the estimation of *A*(*T*) for each realization of the simulated process, then averaging the logarithm of the AFs so that an average estimation of *α* is obtained when we apply the linear regression.

### 3.3. Detrended Fluctuations Analysis (DFA)

Detrended fluctuation analysis (DFA) was originally proposed as a technique for quantifying long-range correlations [11]. For our purposes, this method is applied to the sequence of intervals, as proposed in [9]. After mean value subtraction, the intervals sequence {*I*_*i*_} = (*t*_*i*+1_ − *t*_*i*_) of length *N* is transformed by running summation. The resulting sequence is divided into *M* nonoverlapping blocks of *K* samples (*M* = *N*/*K*). A linear trend is computed for each block by means of linear least-squares fit and removed from the samples in the block.

The variance of the detrended series is computed for each block and the average of these variances is the DFA measure *F*(*K*) relative to block size *K*. For fractal series, *F*(*K*) varies as a power-law: *F*(*K*) = *K* ^2−*α*^, where *α* is the same fractal coefficient obtained with the other methods we have previously discussed. As for the implementation, we exploited a modified version of the algorithm presented in [12]. 

As in the previous cases, we performed the estimation of *F*(*K*) for each realization of the simulated process, then we average the logarithm of the fluctuations in order to obtain an average estimate of *α* by linear regression with *K* ≥ *K*_0_ (where *K*_0_ is manually selected for maximizing the linearity of the fluctuation increase).

## 4. Results

As described in the method section, a large set of simulations of interevent intervals was generated by means of Monte Carlo (MC) sampling from biexponential *pdfs* (generated accordingly to single-synapse data) [4, 6]. In these MC trials we varied the area of the fast exponential component *a*_*f*_ using four values for this free parameter: 2%, 15%, 50%, and 85% [4]. 

**Figure 1:**
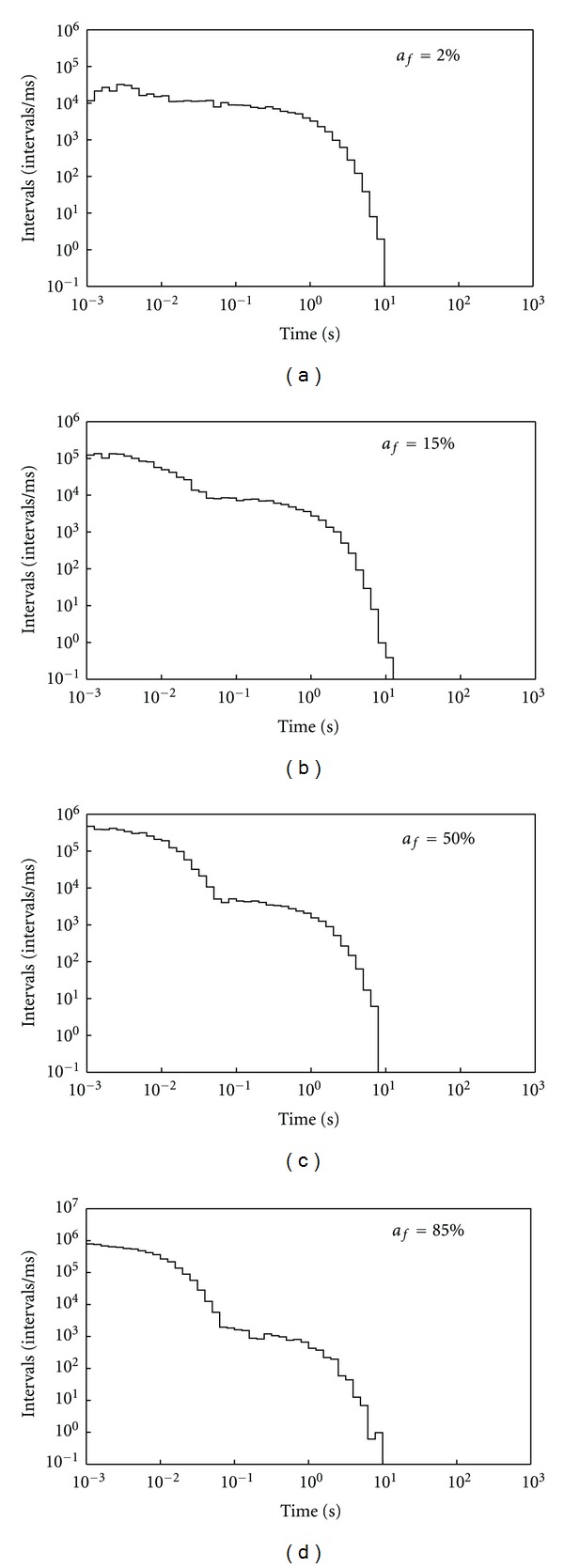
Log-binned histograms of interevent intervals from simulated quantal release series (see Section 2). Each bin-content has been normalized for the corresponding bin-width. The sets of intervals were generated by means of Monte Carlo sampling methods from biexponential probability density distributions with *a*_*f*_ = 2, 15, 50 and 85%.

Figure 1 shows the log-binned histograms for these data sets. As the free parameter *a*_*f*_ increases, the intervals distributions show two clear humps, where the hump on the left side of the *x* axis reflects a larger number of clustered high frequency intervals. It is worth noting that, since the histograms are log-binned, the bins which lie far from the *y* axis are broader than the one on the lie near to it, so in this representation the slow frequency component is over-stressed, altering the visual perception of the contribution of the fast and slow components in these graphs.

Starting from these sets of random intervals, different families of fractal-rate point processes were generated as described in detail in *Methods* section. To generate these simulated events we used an *α* value equal to 0, 0.5, 1, 1.5, and 2. Using these simulated data-sets, three analysis methods were tested: the periodogram (PG), the Allan factor (AF), and the detrended fluctuation analysis (DFA). The goal was to evaluate possible variation in the accuracy of our estimates according to the input parameters: the *α* itself, which represents the fractal feature of the point process, and the *a*_*f*_, which modifies the degree of “bumpiness” of the interval distribution. Another important parameter is *N*, the sample size. When the simulations mimicked the most probable physiological condition, the one found with single-synapse recordings (*a*_*f*_ = 15%; *α* ≈ 1), all methods provided high accuracy in estimating the fractal exponent chosen at simulation time. The sample size *N* of these sequences was kept constant and equal to 10.000 samples.

**Figure 2:**
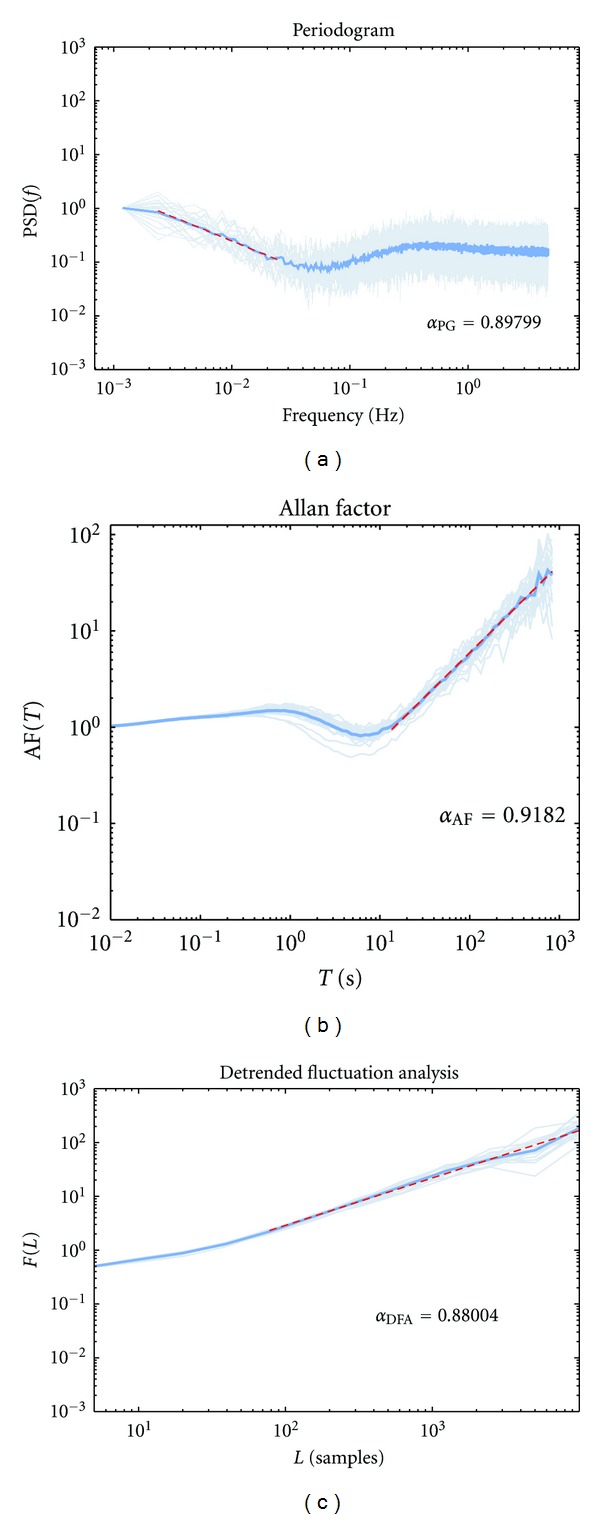
The three methods used for the quantification of the fractal exponent *α* on simulated release series (simulations with *α* = 1 and *a*_*f*_ = 15%). (a) Periodogram (PG), (b) Allan factor (AF), and (d) detrended fluctuation analysis (DFA). Pale-blue lines represent single realizations (*n* = 20); thick blue lines represent the average of measures; red dashed lines are fittings in the selected range. Notice how the variation between different trials is quite limited.

This result is illustrated in Figure 2, where the three methods are graphically compared. For each method, the measures PSD, *A*(*T*), and *F*(*K*) are shown for each realization of the simulated process (pale-blue lines). Over these sets of measures, it is also graphed the average (computed after logarithmic transform) on which the ultimate fractal exponent estimated $\hat{\propto }$ is performed (blue line). Red dashed lines in the plots indicate linear fitting on doubly logarithmic scale of the relative average measures.

To test for the influence of the fast exponential component of the interval distributions on the detection and correct quantification of the power-law behavior, release series were generated with parameter *a*_*f*_ between 2% and 85% (2%, 15%, 50%, and 85% values), and parameter *α* between 0 to 2 (0, 0.5, 1, 1.5, and 2). For these general tests the sample size was kept constant (*N* = 10^4^).  Table 1 shows the numerical results of this performance evaluation.

**Figure 3:**
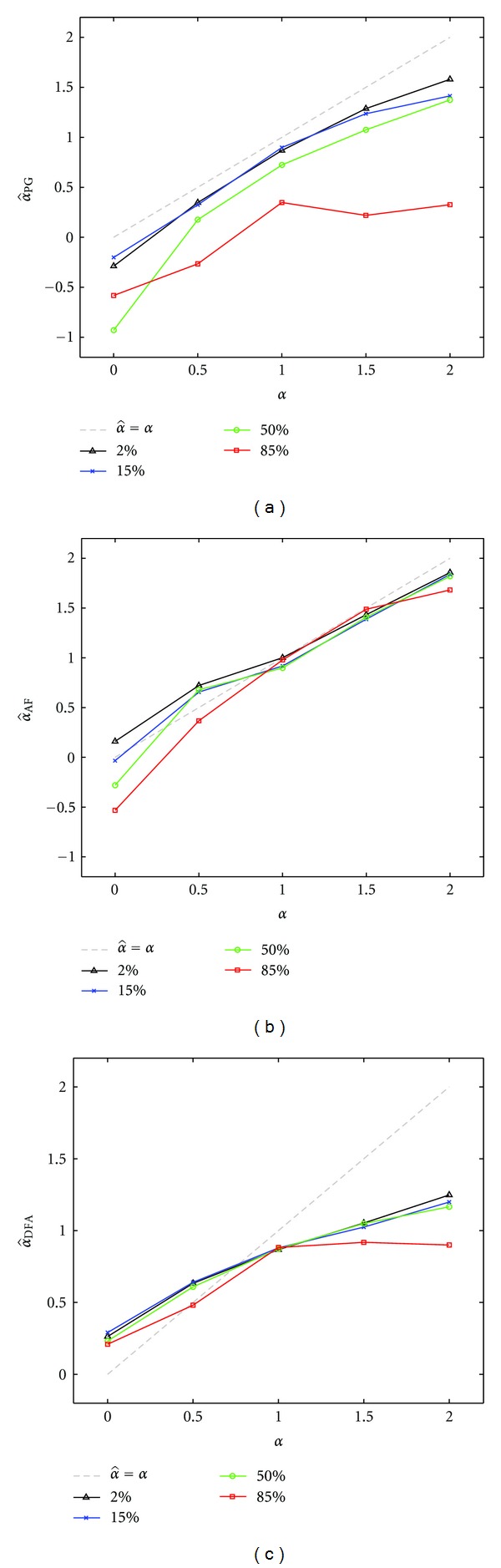
Graphical comparison of the performance of PG, AF, and DFA methods (same numerical results of Table 1). Colored lines, with different marker shapes, refer to values of parameter *a*_*f*_ (as explained in the legends). Gray, dashed line shows the intercept of the graph, that is, the ideal estimated result. AF provides the most reliable method for quantifying the fractal exponent, given its low sensibility to variations of the *a*_*f*_ parameter and to an almost linear behavior as a function of the expected value of *α*. Notice how DFA estimates diverged from the input value when the fractal coefficient is increased above *α* = 1.

For achieving a more direct comparison between the different methods evaluated here, some summary graphs were generated as shown in Figure 3. The values for the parameters are the same as those presented in Table 1. As already evident at first glance (Figure 3), all methods here used are sensitive to variation in the parameter *a*_*f*_ and have different errors estimate when *α* increases from 0 to 2.

Considering the whole range of *α* values, AF seems to be the most reliable method for precisely quantifying the fractal exponent of these release series. DFA performance is strictly non-linear as *α* increases, albeit it gives the most reproducible result for *α* = 1 over different areas of the fast exponential component. The count-based PG has great sensitivity to the interval distribution, providing the worst performance with *a*_*f*_ = 85%.

**Figure 4:**
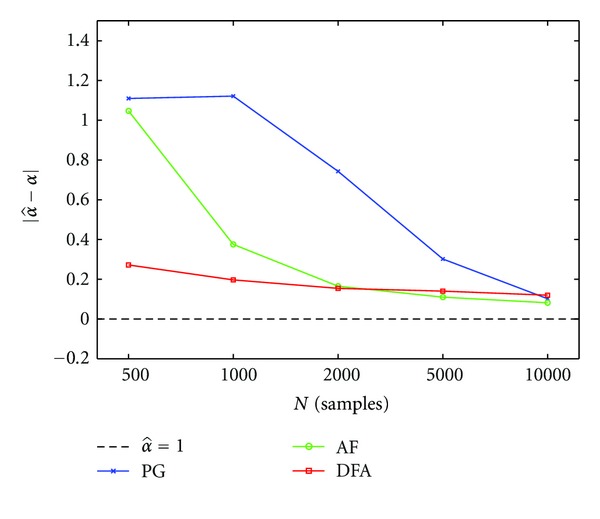
Estimates of the fractal exponent using PG, AF, and DFA methods, evaluated as a function of sample size. Error in the estimate is expressed as the difference between the estimated value and the expected value, taken as absolute value. This test is limited to the simulations with parameters *α* = 1 and *a*_*f*_ = 15%, the case assumed as most physiological. Sample size is varied as follows: *N* = 500, 1000, 2000, 5000, 10000.

We then evaluated the influence of the sample size *N* on the output estimates. This was done in consideration of the fact that in most cases single-synapse recordings data sets are characterized by a limited sample size [4, 6].  Figure 4 illustrates how the error decreases when the sample size is increased from 500 to 10^4^ samples. This analysis was limited to a single combination of parameters (*α* = 1 and *a*_*f*_ = 15%), the most probable case based on our electrophysiological results. The error was computed as the difference between the estimated fractal exponent value and its expected value, taken as absolute value (Figure 4, results for PG, AF, and DFA in different colors).

Our results showed that the PG method seems much more sensible to a reduction in the sample size than the AF and DFA. While the DFA is the least affected of the three methods, with a rather stable error over a range of *N* comprised between 500 to 10.000 samples, the estimates of the fractal exponents obtained using the PG and AF methods become less reliable for *N* below 5000 and 1000 events, respectively. This poor performance of the PG, when the sample size *N* is small, is probably ascribable to the fact that, at odd with the other methods, the DFA uses a sequence of intervals rather than a counting sequence.

## 5. Discussion

In the present paper we have used the gold standard methods for the study of fractal processes, that is, the periodogram, the Allan factor, and the DFA method, to test their sensitivity and reliability in detecting the 1/*f* power law behavior in the rate spectrum of spontaneous synaptic quantal releases series [2, 5, 9].

Since our goal was to apply these tests to single synapse data, which are characterized by small a sample size, multi-exponential interevent interval distributions and large intersynaptic rate variability, we generated large collections of simulated series of release events characterized by features which were designed to match those found experimentally [4].

To this aim, we combined Monte Carlo sampling methods from biexponential probability density functions, with characteristics mimicking the observed experimentally (*a*_*f*_ = 2–85%; *N* = 500–10.000), with an integrate-and-fire model to generate different fractal-rate processes (parameter *α* was set between 0 to 2). In the evaluation of the input parameters, the periodogram was always used as a reference.

Besides its ability to represent the 1/*f* behavior, in the presence of a stationary signal, PG also provides a complete representation in the frequency domain. Our results show that the periodogram, despite its reduced sensitivity for small sample sizes *N*, provides a good estimate of the input alpha parameter. Based on the analysis presented here, in the presence of hyperexponential distributions, the most reliable and sensitive method to extract the fractal coefficient *α* is the Allan factor. The AF was clearly the best of the three tested methods, capable of extracting with high degree of precision the input fractal coefficient *α*. For this reason, it would be the method of choice for the analyses of single-synapse data, which are characterized by small and very small *N*, and to make comparisons between different experiments. On the contrary, the DFA method was found to be less reliable, essentially because it showed a sort of saturation of the output values for increasing input *α* values above 1 (Figure 3). This might limit its application to real data, since synaptic populations are composed of synapses whose developmental profile, morphology, and activity are very heterogeneous. Because of this, the fractal coefficient alpha is expected to display large variation among different synapses which the DFA method cannot clearly capture. On the other hand, DFA is apparently less sensitive to variation in the sample size. This presumably depends upon the fact that this method uses a sequence of intervals rather than a counting sequence. This suggests that the length of time epochs becomes less important, therefore even a small number of intervals might be sufficient to estimate properly *α* with the DFA method.

In summary, based on these considerations, the AF method is the method of choice for the quantitative analysis of fractal behavior at single-synapses, a conclusion which is perfectly in line with the DFA description and its applications to a large number of conditions [9].

In relation to future goals, the results of this work will be used to further evaluate the reliability of the assertion that data from single-bouton recordings [4] indeed follow a 1/*f* behavior [5]. Fractal behavior at individual neuromuscular junctions have been previously reported and thoroughly demonstrated [2]. Unfortunately, the neuromuscular junction is a very large compartment, composed of a multitude of active zones and millions of available vesicles, filled with neurotransmitter molecules [13]. In these conditions voltage and ionic fluctuations occurring inside the synapse might affect many quanta simultaneously and the related diffusional processes might shape and correlate in complex ways spontaneous release sequences. The story is clearly very different at central synapses, which in most cases are small, self-contained compartments with a single active-zone and a small number of readily available quanta data (for the fine morphology of hippocampal synapses from which the data used here were derived see [4]). Therefore, the nature of possible correlation events might be very different at central synapses. In this respect, standard whole-cell recordings from post-synaptic neurons, which sample many synapses simultaneously [2–4], are not enough and single-bouton recordings, which sample from just one synapse, from one active zone at the time, would be preferable [4, 6]. The possibility that the release process at one synapse follows the 1/*f* power law [5] clearly indicates that this behavior is synapse-autonomous and involves just one active zone [4, 6].

Although the underlying mechanism is far from clear, such synaptic memory might relate to some specific structural or functional features of the active zone of central synapses. At the present time, despite the lack of a better knowledge about the molecular and functional organization of the synaptic release sites precluding any deeper mechanistic understanding [14, 15], the analytical methods might soon provide novel insight into these processes.

## Figures and Tables

**Table 1:** Numerical results of fractal exponent estimations with PG, AF, and DFA methods. Simulated release series are characterized by parameters: *N* = 10^4^, *a*_*f*_ = 2%, 15%, 50%, 85%, and *α* = 0, 0.5, 1, 1.5, 2.

*α*	PG	AF	DFA
	*a*_*f*_ = 2%		

0	−0.288	0.160	0.263
0.5	0.347	0.721	0.633
1	0.869	1.001	0.868
1.5	1.287	1.433	1.053
2	1.580	1.856	1.248

	*a*_*f*_ = 15%		

0	−0.201	−0.034	0.291
0.5	0.324	0.656	0.640
1	0.898	0.918	0.880
1.5	1.236	1.388	1.025
2	1.414	1.838	1.199

	*a*_*f*_ = 50%		

0	−0.929	−0.281	0.232
0.5	0.176	0.678	0.609
1	0.723	0.898	0.872
1.5	1.074	1.408	1.049
2	1.374	1.818	1.166

	*a*_*f*_ = 85%		

0	−0.582	−0.532	0.210
0.5	−0.265	0.367	0.481
1	0.348	0.979	0.883
1.5	0.219	1.488	0.919
2	0.326	1.681	0.900
